# A Century of Vaccination

**Published:** 1896-11

**Authors:** 


### A Century of Vaccination.

A hundred years have passed away since Jenner's first successful vaccination on May 14, 1796. Jenner's brilliant idea, pondered over for more than twenty-five years, was that small-pox might be abolished by the universal adoption of vaccination. Others had vaccinated before Jenner, but he was the first to rouse the civilized world to take an active interest in the subject; and we must not forget that vaccination was not the outcome of laboratory experiments, but a practice resting upon a common experience in many countries of Europe, not to mention Mexico and Persia, that dairy maids and others who had "sore hands" from milking cows affected with cowpox were afterward found to be protected against small-pox. The present time invites us to review the progress of vaccination in this and other countries, with the concomitant alterations in the mortality from small-pox, and to make an attempt to gather into a few lines the teachings of a century.

What was the average yearly mortality per million living from small-pox, which we will throughout call the mean rate, during the last century? In the large cities it was 3,000, and in the whole nation it was at least over 2,000. This is the mean rate. During some years the mortality rose as high as 5,000 or 6,000 per million, and even higher. Now we find a rapid fall of small-pox in every country which we examine as soon as vaccination became common. The fall was abnormal in one respect, because the adult population of Europe then consisted largely of survivors of small-pox in childhood. This fall is closely connected with the rise of vaccination in every country separately. Thus, in Sweden, during the 28 years 1774–1801, before vaccination the mean rate is 2,045; during 15 years 1802–16 of permissive vaccination it is 480, and during 77 years, 1817–94, of compulsory vaccination it is 155. During the last ten years the mortality is insignificant. In England in the last century the mean rate was over 2,000, according to able statisticians; during 12 years of permissive vaccination, 1838–53, the mean rate is 417; during the succeeding 18 years of enjoined vaccination the rate sinks further to 154; and the mean rate since the epidemic of 1871–72 under enforced vaccination is only 53, that is, for the 22 years 1873–94; while for the 10 years 1885–94 the mean rate is 26. The law really enforcing vaccination dates only from 1871. In Prussia, vaccination was encouraged only, not enforced on all children, till the law of 1874, after which all children born in the German empire were required to be vaccinated by the end of the second year of age, and all school children to be revaccinated. Well, the permissive era yields a mean rate of 309, but the 18 years, 1875–92, have a mean rate of only 15, and during the last 10 years of this the deaths from small-pox in Prussia average only seven per million yearly. In Austria vaccination is not compulsory yet. Austria's mean rate during the 35 years, 1847–82, is 58C. One more example of a country still without compulsory vaccination, namely, Belgium. The mean rate for the ten years, 1875-84, is 441. This, again, is a rate resembling that of England or Sweden in the permissive era. In fact, we can say with confidence what the vaccination law is in any country from a mere inspection of the small-pox mortality for some years. Italy has followed Germany since 1888. Vaccination in infancy was then made universally compulsory, and also the revaccination of all children attending public schools. The mean rate per million in the chief townships during the nine years previous to the law as put in practice is 440—just as we might have expected; the average during the five years, 1890–94, is 100; the average for all Italy during this latter period is 110.

We are dealing now with large countries and vast populations, and we are considering the small-pox mortality alone, apart from the question of the vaccinated or non-vaccinated condition of those who died. Examples enough have been given to show the remarkable uniformity that exists in the death rates of various countries, according to the state of the law in those countries. No other cause than vaccination can account for this. It cannot possibly be improved sanitation that has caused the remarkable changes in the mean death rate above given, for more than one reason. Compare Prussia with Austria. There is a sudden and striking change in the small-pox death rate of Prussia in the period succeeding the law of 1874. Now Austria shows no such change in the death rate, and Austria is still without compulsory vaccination, while Austria has participated in the sanitary improvements of the age. And, on the other hand, Prussia did not suddenly jump into an ideal sanitary condition between 1870 and 1880. But further, the reduction in small-pox mortality has not affected all ages alike, whereas improved sanitation does affect all ages alike. Again, it is absurd to talk of a natural decline of small-pox, as plague has declined and vanished, from this country at least, when we observe the virulence of small-pox in local outbreaks, and when we think of the very large mortality which countries like Spain and Russia still show, countries where there is very little vaccination. Here are the small-pox death rates per million living, for the single year 1889, in the following provinces of Spain: Almeria, 3,080; Murcia, 2,670; Coruña, 1,230; Malaga, 1,340; Cadiz, 1,330; Cordoba, 1,400. The rate for Germany is four for the same year.

What are the lessons which a hundred years' experience of the practice of vaccination by various nations taught mankind? First, we know that the rapid spread of this practice was partly due to an erroneous idea of the early vaccinators, Jenner himself included, namely, that one vacccination in childhood was sufficient to pretect for life. Secondly, we know that the most rigidly enforced vaccination in infancy alone in any country is insufficient to prevent severe outbreaks of small-pox. Thirdly, primary vaccination vastly reduces the mortality from smallpox, but it also shifts the incidence of this mortality from childhood to adult life. The natural susceptibility toward small-pox sinks of itself from the first year of life till the end of childhood or the beginning of puberty, when it is lowest, after this it rises gradually with age. More adults now die of small-pox than in the early days of vaccination.

But statistics teaches us that a successful revaccination during school age completely alters the situation, and renders a person safe for life against small-pox, with rare exceptions. Even a survived attack of small-pox does not absolutely protect against death from a second attack. Germany over twenty years ago acted upon these well known truths, and by the law of 1874, enforced both the compulsory revaccination of all school children and the vaccination of all childred before the age of two years. And in Germany small-pox epidemics are abolished, and most of the few cases which occur are on the boundaries of the empire.—*British Medical Journal.*

